# Rab GTPases and the Rabopathy framework: from synaptic trafficking to neurodegeneration via LRRK2-driven pathology

**DOI:** 10.1186/s40035-026-00596-8

**Published:** 2026-11-09

**Authors:** Linan Zha, Jing Xu, Xiaoling Zhu, Qikun Tu, Chunwei Zhong, Linshi Zhan, Mojiao Zhang, Dongshan Zhou, Xifeng Wang, Jianhang Wu, Fuzhou Hua, Xilong Guan

**Affiliations:** 1 Department of Anesthesiology, Nanchang University Affiliated Yingtan Hospital, Yingtan, 335099 China; 2 Key Laboratory of Anesthesiology of Jiangxi Province, Nanchang, 330006 China; 3 Department of Anesthesiology, The First Affiliated Hospital, Jiangxi Medical College, Nanchang University, Nanchang, 330006 China; 4 Department of Anesthesiology, The Second Affiliated Hospital, Jiangxi Medical College, Nanchang University, Nanchang, 330006 China

**Keywords:** Rab GTPases, LRRK2, Rabopathy, Membrane trafficking, Autophagy-lysosome pathway, Neurodegeneration, RAB39B

## Abstract

Neurodegenerative diseases such as Alzheimer's disease (AD), Parkinson's disease (PD), and Huntington's disease (HD) show significant differences in their clinical manifestations, but they all seem to share a common upstream problem: malfunction in the membrane transport controlled by Rab GTPases. This review introduces the concept of Rabopathy, a framework that connects developmental synaptic maturation defects with late-onset protein aggregation. The LRRK2–Rab phosphorylation axis is emerging as a potential common amplifying mechanism across these disorders, with the strongest mechanistic support in PD and growing implications in AD and HD. When Rab substrates are excessively phosphorylated at specific sites, vesicular transport is impaired or arrested, leading to progressive depletion of the reserve pool of synaptic vesicles and the surface pool of receptors, and severe compromise of the autophagy-lysosome flux. The failure of distinct Rab sub-networks also produces disease-specific signatures, including Rab5-driven endosomal congestion in AD, Rab35-mediated α-synuclein secretion in PD, and Rab11/Rab7 trafficking collapse in HD. Restoration of Rab flux may be a therapeutic strategy for neurodegenerative diseases, which can be achieved through calibration of LRRK2 activity, modulation of selective Rab subtypes, and rescue of the trafficking network at the systems level.

**Graphical Abstract:**

**Figure:**
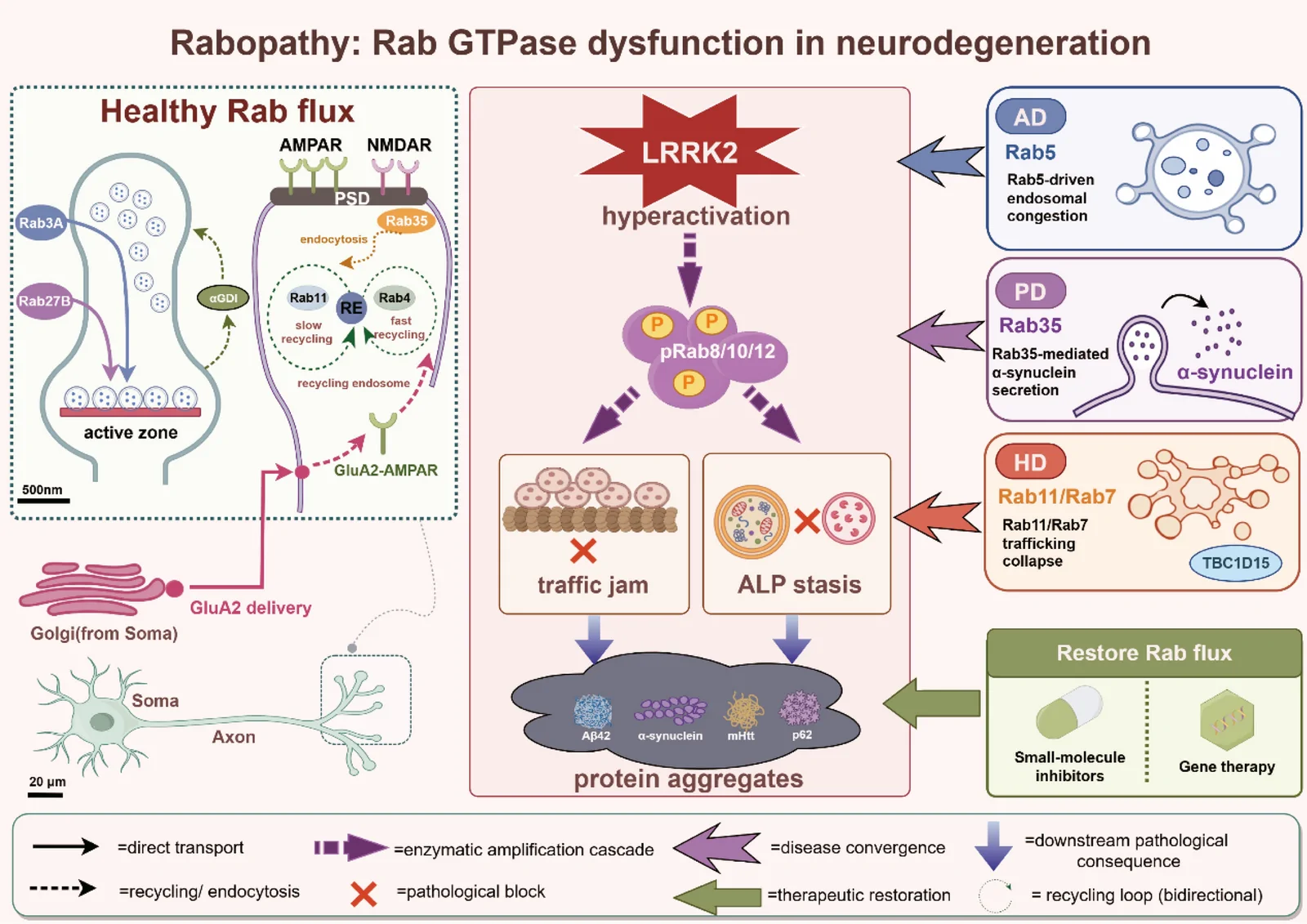

## Introduction

In Alzheimer's disease (AD), Parkinson's disease (PD), Huntington's disease (HD), and neurodevelopmental conditions associated with Rab dysfunction, the spatiotemporal precision of intracellular membrane trafficking is progressively lost. With decline of this precision, synaptic proteostasis and organelle stability are compromised, which eventually leads to neuronal dysfunction and death. The trafficking network is coordinated by the Rab small monomeric GTPases, a family of roughly 70 members in mammals that control key steps of the vesicle life cycle, from budding and cytoskeletal transport to tethering and fusion [1–3]. By switching between an active guanosine triphosphate (GTP)-bound state and an inactive guanosine diphosphate (GDP)-bound state, Rab proteins recruit specific effector complexes, which establish the identity of individual membrane compartments and guide cargos through the endocytic or secretory pathway [1, 3]. Regulation of the trafficking is particularly demanded in highly polarized neurons, since neurotransmitter receptors, synaptic vesicle components, and neurotrophic factors must all be delivered in a tightly coordinated manner to maintain synaptic plasticity [4, 5].

Rab dysfunction is now increasingly regarded as a convergent mechanism of neurodegeneration. Recently, leucine-rich repeat kinase 2 (LRRK2), a major genetic determinant of PD that also modifies AD risk, has been identified to function as a kinase hub and phosphorylate a defined subset of Rab GTPases at specific sites [6]. Once the Rab substrates, most notably Rab8, Rab10, Rab12, and Rab5 which is recently identified as a direct LRRK2 substrate, become hyperphosphorylated, their interactions with canonical effectors and GDP dissociation inhibitors (GDIs) are disrupted, blocking vesicular transport at defined checkpoints. This gives rise to enlarged early endosomes and autophagosome-lysosome blockade, which are early cytopathological features of AD and PD [6–12].

However, these findings remain to be integrated into a pathogenic framework, because LRRK2-mediated phosphorylation events and individual cases of Rab dysfunction have so far been characterized largely within isolated disease contexts; it therefore remains unclear how early trafficking defects progress along a developmental trajectory toward late-life neurodegeneration, and how apparently disparate pathways converge at the mechanistic level. RAB39B exemplifies this spectrum well, since its mutations give rise to both X-linked intellectual disability and early-onset PD, suggesting that early-life defects in ionotropic glutamate receptor maturation and secretory transport lead to a latent vulnerability that becomes manifest only under cumulative stress [13, 14]. At the same time, the developmental perspective alone cannot account for the whole picture, as Rab dysfunction also reaches beyond neurons. In astrocytes and microglia, endocytic and phagocytic trafficking supports the clearance of amyloid-β (Aβ) and α-synuclein aggregates [15–18], and both processes depend inherently on Rab-regulated vesicle transport.

On this basis, the present review proposes the term Rabopathy as a unifying pathogenic framework in neurological disorders marked by progressive dysfunction of Rab GTPase-regulated membrane trafficking. Under the current circumstances, this concept is mainly applicable to the neural development and neurodegenerative diseases of the nervous system, including but not limited to AD, PD, and HD. Although Rab GTPases are ubiquitously expressed in all eukaryotic cells and support membrane transport, due to the extreme polarity of neurons and their extremely high transport demands, they exhibit selective vulnerability to Rab dysfunction. The framework therefore focuses specifically on the nervous system, where axonal length, synaptic density, and activity-dependent membrane turnover impose unique physical and energetic constraints that further amplify the pathological consequences of Rab dysfunction. Accordingly, the term covers any Rab GTPase whose dysregulation compromises synaptic, endolysosomal, or autophagic flux, rather than being restricted to LRRK2-phosphorylated substrates.

Taken together, Rab proteins and their regulatory partners constitute an emerging therapeutic target. Since Rab signaling cascades are interconnected, pharmacological strategies aimed at restoring network homeostasis, such as LRRK2 inhibitors or Rab-specific modulators, may theoretically prove more effective than targeting single protein aggregates in preclinical models [6, 19]. In this review, we integrate mechanistic insights with clinical and genetic evidence to construct a systematic Rabopathy framework. We first delineate Rab functions in healthy neuronal trafficking. Then we dissect the LRRK2-Rab phosphorylation axis as a shared amplification mechanism, describe cross-disease convergence on the autophagy-lysosome pathway (ALP) as a shared endophenotype, and review disease-specific Rab network failures in AD, PD, and HD. Finally, we outline therapeutic perspectives within the framework.

## Rab GTPases in healthy neuronal trafficking: core mechanistic foundations

**Fig. 1:**
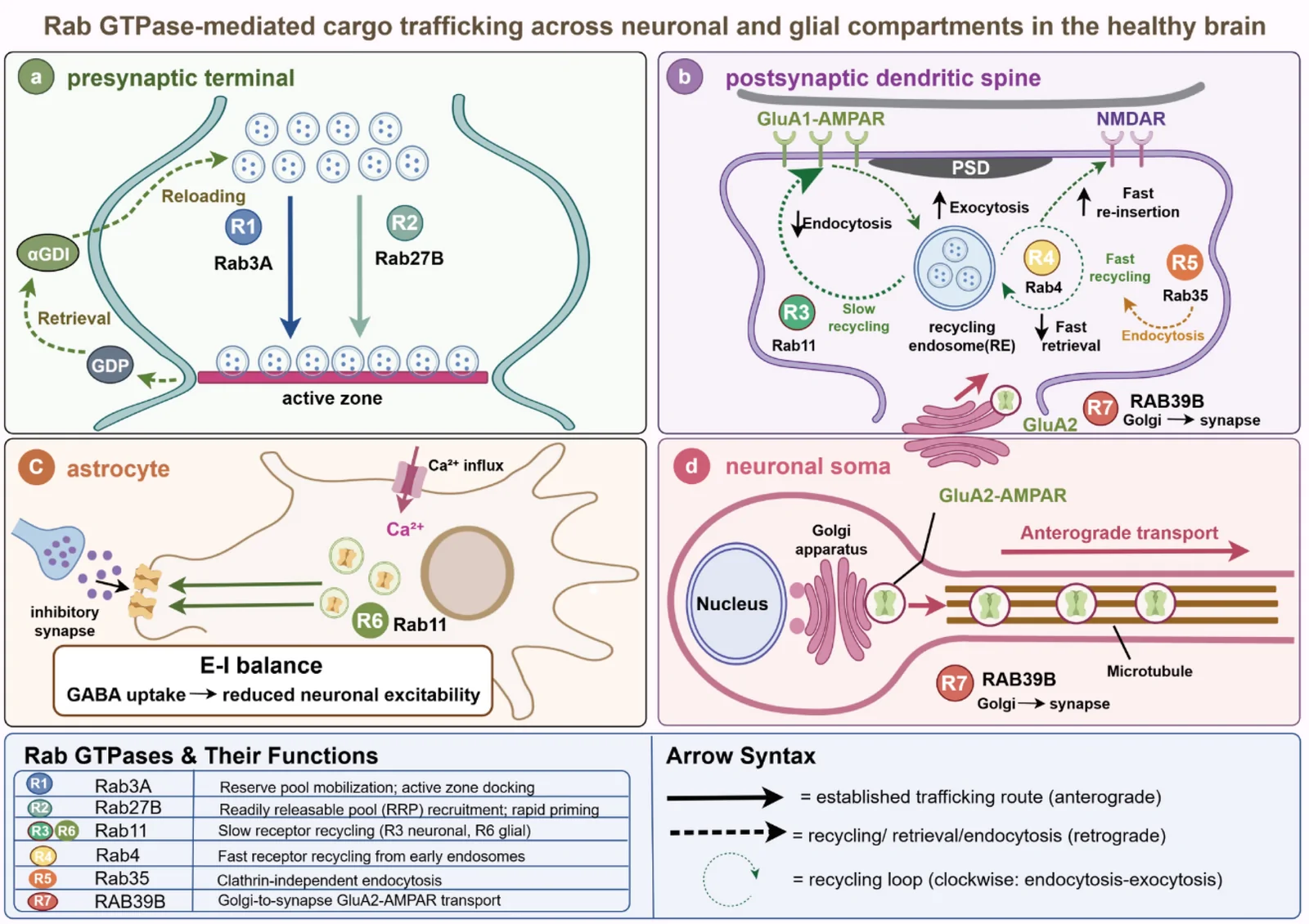
Rab GTPase-mediated cargo trafficking across neuronal and glial compartments in the healthy brain. Schematic overview of Rab-mediated membrane trafficking pathways that support synaptic transmission and plasticity. **a** In the presynaptic terminal, the synaptic vesicle reserve pool is maintained by Rab3A (R1), whereas recruitment into the readily releasable pool at the active zone is governed by Rab27B (R2). αGDI (*GDI1*) serves as the cytosolic recycling station for GDP-bound Rabs. **b** In the postsynaptic dendritic spine, GluA1-AMPAR and NMDAR surface delivery is jointly coordinated by Rab11 (R3)-dependent slow recycling and Rab4 (R4)-driven fast recycling from recycling endosome, while clathrin-independent receptor endocytosis is facilitated by Rab35 (R5). **c** In the astrocyte, surface expression of GABA transporter (GAT) is regulated by Ca^2^⁺-dependent Rab11 (R6) trafficking, through which GABA uptake and the excitation-inhibition balance are in turn modulated. **d** In the neuronal soma, Golgi-to-synapse transport of GluA2-containing AMPARs is mediated by RAB39B (R7). Established trafficking routes are indicated by solid arrows, while dashed arrows indicate recycling, retrieval, or endocytosis routes; R3 and R6 correspond to Rab11 in neuronal and glial compartments, respectively

Higher brain functions rest on synaptic communication and homeostatic maintenance, which depend on neuronal membrane trafficking. Rab GTPases serve as the molecular executors within this trafficking network, organizing the intracellular space into distinct functional compartments. Through their coordination of neurotransmitter receptor trafficking and synaptic vesicle (SV) recycling, the continuous renewal and activity-dependent adjustment of synaptic components are maintained (Fig. 1).

### Presynaptic vesicle cycling: complementary roles of Rab3A and Rab27B in vesicle pool dynamics

The presynaptic terminal is the most demanding trafficking hub in the nervous system: neurotransmitter release must occur with millisecond precision. Rab3A, the most abundant Rab protein in the brain, is a principal regulator of SV life cycle. Early biochemical studies established Rab3A as a vesicular marker that promotes docking and post-fusion recycling of vesicles at the active zone through interaction with effectors such as Rab3-interacting molecule [20]. This interaction maintains the reserve pool and ensures sustained neurotransmitter release during high-frequency stimulation [21]. In Rab3A-deficient neurons, SVs cannot be mobilized from the reserve pool after repetitive stimulation, and long-term potentiation (LTP) at mossy fiber synapses cannot be induced [22, 23]. These findings indicate that Rab3A maintains the releasable vesicle supply, preventing synaptic exhaustion during sustained activity. Reserve pool mobilization, however, operates on a relatively slow timescale. For synapses responding to transient, high-frequency bursts without depleting the backup supply, a second, faster regulatory layer is required. Rab27B operates at this level by controlling the size of the readily releasable pool [24, 25].

In PC12 neuroendocrine cells, the effector Slp4-a (synaptotagmin-like protein 4a, also known as granuphilin-a) regulates dense-core vesicle exocytosis through interactions with GTP-bound Rab3A, Rab8, and Rab27A [25]. Whether Rab27B recruits a comparable effector scaffold to organize SV capture at central synaptic active zones remains to be established. Within this framework, Rab27B is thought to govern the priming of vesicles already captured within the readily releasable pool, whereas Rab3A mediates mobilization from the more distant reserve pool. Rapid responses to transient stimuli and the maintenance of sustained release are two aspects that must be balanced for normal synaptic function. The former depends on the activation of Rab27B-dependent pathways, while the latter relies on Rab3A to maintain the capacity for reserve recruitment. Although they serve different functions, these two Rab proteins do not operate in isolation but rather coordinate with one another as activity patterns change. As a result, synapses maintain their adaptability regardless of whether they are in a state of sustained firing or brief bursts. Over evolutionary time, overlapping functions have made this division of labor harder to disrupt. Even quadruple knockout of Rab3 family members leads to only a ~30% reduction of release probability [26]. The modest phenotype suggests that presynaptic release is strongly buffered after Rab3 loss. Rab27B and related Rabs may contribute to this buffering, although the compensatory Rab(s) remain to be identified. Developmental or homeostatic compensation may also make the quadruple-knockout phenotype an incomplete measure of the normal contribution of Rab3A. Rab27B remains relevant here because it regulates excitatory synaptic transmission and plasticity [24], but its specific role in rapid sequence processing, working memory and early neurodegeneration requires further study.

There is an interesting paradox in the aging brain. On the one hand, Rab3A expression declines with age. On the other hand, membrane-bound Rab3A protein actually accumulates in the brain [27]. Whether this apparent contradiction reflects a decline in recycling efficiency remains to be established, although age-related changes in the membrane–cytosol distribution of small GTPases have been reported in the aged mouse brain [28]. When recycling fails, Rab3A is trapped on the membrane. The pool of circulating GTPases is then depleted. In the early phase this inefficiency is often masked by redundant mechanisms. Basic signal transduction is maintained and the problem is hard to detect. Age-related Rabopathy may thus begin with a loss of fluidity in the transport cycle rather than a loss of protein. Another interpretation is that the age-related change in Rab3A reflects altered cycling rather than a simple change in abundance. The shift from dynamic cycling to static membrane accumulation may be the earliest detectable sign of failing synapses. It appears before overt neurodegeneration and long before clinical decline.

### Postsynaptic plasticity: the Rab11-Rab4 recycling axis and Rab35-mediated receptor turnover

Receptor transport is governed by a tightly coordinated shuttle system. Its core components are Rab11 and Rab4 [29–31]. The strength of α-amino-3-hydroxy-5-methyl-4-isoxazolepropionic acid (AMPA) receptor (AMPAR) and N-methyl-D-aspartate (NMDA) receptor (NMDAR) synapses depends on the activity-dependent regulation of this system [30, 32]. Two parallel recycling pathways operate within it. One is the slow loop mediated by Rab11. The other is the fast loop mediated by Rab4 [33, 34]. Rab35 runs a distinct trafficking route that controls exocytic secretion and endolysosomal quality control [35, 36]. Under pathological conditions this route is repurposed. Pathogenic proteins such as α-synuclein are then secreted and spread between cells [36, 37].

The Rab11-positive recycling endosome acts as a major platform for receptor delivery. Through interactions with scaffolds such as APPL (adaptor protein, phosphotyrosine interacting with PH domain and leucine zipper), Rab11 ensures that receptors reach the synapse during growth and plasticity [32, 33, 38, 39]. The slow recycling loop depends on actin-based transport with myosin Vb, while the fast loop, driven by Rab4, provides rapid responses to acute stimuli [30]. Rab4 regulates the rapid recycling of GluA1-containing AMPARs from early endosomes, and impaired transfer between Rab4- and Rab11-positive endosomes reduces surface AMPAR levels [31]. Neurobeachin is an additional factor in NMDAR endocytic recycling. It is a highly dynamic component of membrane tubules that extend from Rab4 recycling endosomes and transiently enters dendritic spines in an activity-dependent manner to regulate endocytic recycling and surface re-insertion of GluN2B-containing NMDARs [40]. Rab-mediated receptor trafficking enables activity-dependent synaptic plasticity by redistributing receptors from intracellular pools to the membrane [40]. Impairment of the Rab11 or Rab4 machinery may lead to the loss of activity-dependent remodeling in neurons, resulting in impaired synaptic plasticity. Rab35 exerts functions beyond the postsynaptic compartment. Although essential for neurite outgrowth and myelination during development, Rab35 is associated with the aggregation and prion-like secretion of mutant α-synuclein later in life [41]. Rab4 and Rab11 recycle AMPARs at different speeds. Rab4 drives fast recycling from early endosomes, whereas Rab11 drives slow recycling through the endosomal recycling compartment [30, 31, 33]. Disruption of either pathway reduces surface AMPAR levels [31]. AMPARs can also be anchored at the synapse by PDZ (postsynaptic density-95/discs large/zonula occludens-1)-domain proteins such as GRIP1 (glutamate receptor-interacting protein 1) and PICK1 (protein interacting with C kinase 1), independently of Rab-mediated vesicular transport [33]. It should also be noted that the majority of the mechanistic insights in this section derive from hippocampal LTP models, and their generalizability to other brain regions and forms of plasticity remains to be determined.

The dual nature of Rab35 indicates that the neuronal trafficking network that evolved for high-efficiency cargo transport may also play a role in distributing toxic cargo under stress. In this sense, Rabopathy often arises not from a simple loss of transport mechanisms, but from context-dependent repurposing of the machinery while it remains intact.

### Neuron-glia communication: Rab11-mediated metabolic coupling

In addition to neurons, the metabolic and signaling coupling between neurons and glia also contributes to cognitive function. In astrocytes, Rab11-mediated trafficking of γ-aminobutyric acid (GABA) transporters (GATs) regulates transporter surface expression in response to Ca^2+^ signaling, thus modulating GABA uptake and neuronal excitability [42].

The astrocytic Rab11-GAT axis extends the Rabopathy framework beyond neurons. Ca^2^^+^ influx in astrocytes triggers Rab11-dependent rapid endocytosis of GATs, and the resulting increased synaptic GABA levels contribute to neuronal silencing and suppression [43]. Brain-derived neurotrophic factor (BDNF) increases GAT-1 surface expression through tropomyosin receptor kinase B (TrkB)-mediated signaling, although how much Rab11 contributes to GAT-1 trafficking remains to be defined [44]. Disruption of this astrocytic Rab11–GAT pathway may lead to epilepsy and hypersensitivity phenotypes with cognitive impairment. Rabopathy can therefore be considered to implicate the neuro-glial system, in which neurons can no longer maintain the excitation-inhibition balance when astrocytic trafficking of GATs fails.

In the astrocytic Rab11-GAT cycle, Rab11 switches to the GDP-bound state after membrane fusion. All Rabs follow this pattern. αGDI (the α isoform of GDI, encoded by *GDI1*) extracts GDP-bound Rabs, including Rab11, from the membrane and stabilizes them in the cytosol for redelivery [45, 46]. When αGDI function is compromised, as occurs in X-linked intellectual disability-associated mutations, Rab proteins are trapped on membranes and cannot re-enter active transport cycles, halting both neuronal and glial membrane trafficking [47, 48]. In other words, the astrocytic Rab11-GAT axis collapses not due to a primary defect in Rab11 itself, but due to the failure of the recycling infrastructure that sustains its dynamic cycling. This points to a key principle of the Rabopathy framework, that the vulnerability of any individual Rab-dependent pathway is magnified once the global trafficking infrastructure is compromised, and that cognitive deficits emerge from the loss of Rab flux across the neuro-glial system. Notably, human *GDI1* mutations also show considerable phenotypic heterogeneity, suggesting that additional genetic or environmental modifiers shape the severity of Rabopathy in different individuals.

### RAB39B: a molecular bridge between neurodevelopmental disorders and neurodegeneration

A unique longitudinal view of how trafficking defects shape cognition across the lifespan is provided by RAB39B. Acting as a molecular gatekeeper of the Golgi apparatus, RAB39B is responsible for proper maturation and delivery of GluA2-containing AMPARs to synapses [49–51]. The developmental role of RAB39B is further supported by its upstream activator DENND5B (DENN domain-containing 5B), whose de novo mutations cause cognitive impairment, epilepsy, and white matter defects [52, 53].

Loss of *RAB39B* initiates a state of synaptic developmental arrest, in which a population of immature synapses enriched in Ca^2^⁺-permeable AMPARs (CP-AMPARs) persists, resulting in a hyperexcitable network that lacks the refinement required for adult cognition [49, 54, 55]. This maturation failure accounts for early-onset intellectual disability and autism, while the persistent CP-AMPARs also increase the susceptibility to calcium stress and excitotoxicity, providing a mechanistic link to the early-onset parkinsonism observed in these patients. RAB39B thus stands as one of the clearest human genetic examples showing that a developmental trafficking defect can cause a lifelong vulnerability that eventually culminates in neurodegeneration.

Another feature of RAB39B is dosage sensitivity. Both deficiency and excess of RAB39B disrupt neuronal branching and synaptic density [56]. On this basis, therapeutic strategies cannot rely simply on overexpressing Rab proteins, but must instead restore the precise physiological levels. Importantly, the *RAB39B*-deficiency phenotype is reversible, as pharmacological inhibition of CP-AMPARs with 1-naphthyl acetyl spermine (NASPM), a selective CP-AMPAR antagonist, restores dendritic spine dynamics in *RAB39B*-deficient mice [49, 54]. This proof-of-principle study suggests that even lifelong Rabopathy is a functional state that can be corrected once the underlying trafficking imbalance is resolved. It should nevertheless be noted that, although NASPM rescues spine dynamics in mouse models, its selectivity for CP-AMPARs remains incompletely understood, and that *RAB39B* mouse models do not recapitulate the full phenotypic spectrum seen in human patients. Therefore, patient-derived cellular models are needed to validate the therapeutic strategies.

## The LRRK2-Rab phosphorylation axis: a core pathogenic engine

**Fig. 2:**
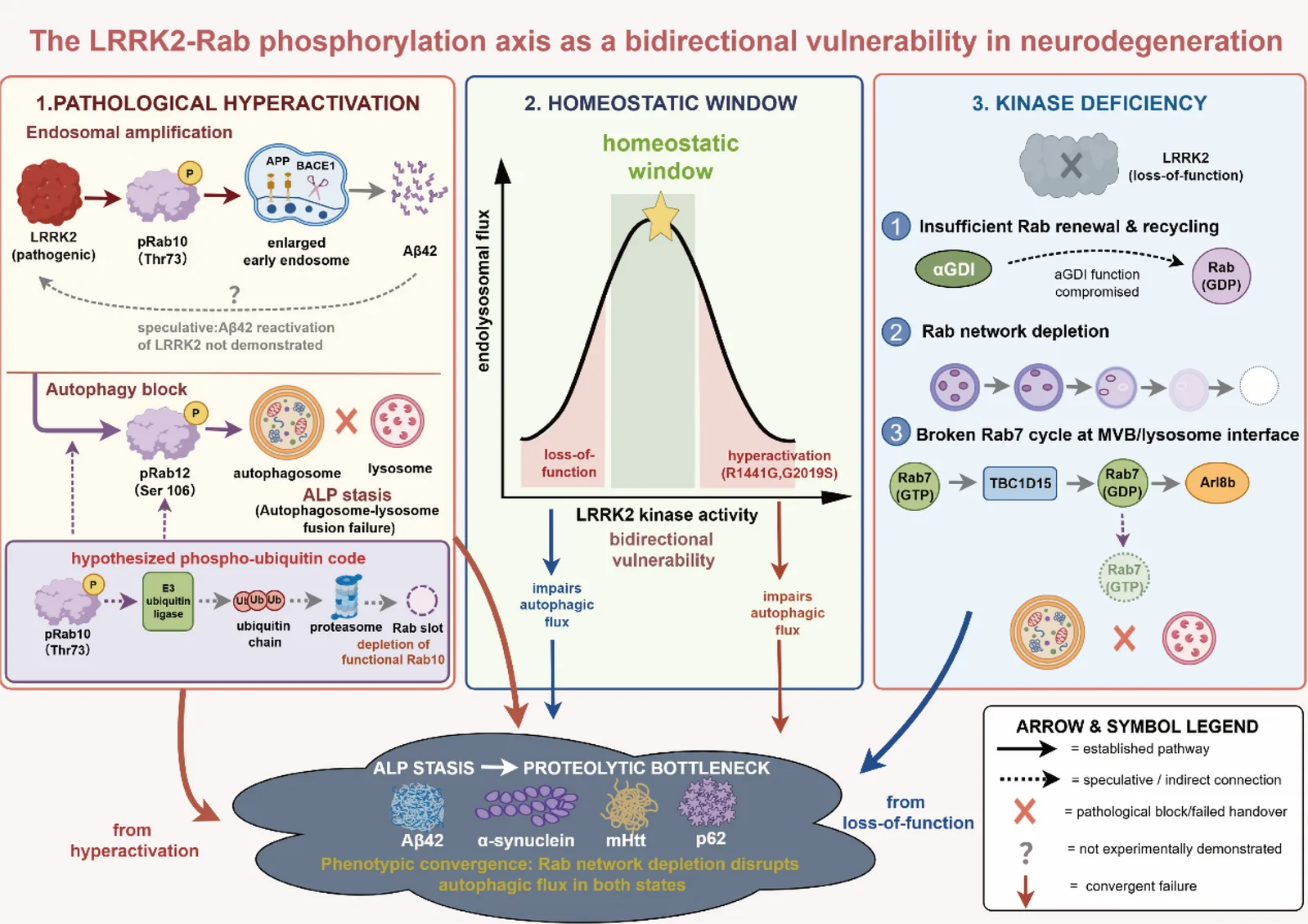
Bidirectional vulnerability of the LRRK2–Rab phosphorylation axis in neurodegeneration. (1) Pathological hyperactivation. Pathogenic LRRK2 phosphorylates Rab10 at Thr73. The resulting pRab10 is proposed to associate with enlarged early endosomes, where amyloid precursor protein (APP) encounters β-site APP-cleaving enzyme 1 (BACE1), facilitating Aβ42 generation. Whether Aβ42 reactivates LRRK2 remains speculative. Concurrently, Rab12 phosphorylation at Ser106 contributes to autophagosome–lysosome fusion failure and autophagy–lysosome pathway (ALP) stasis. Inset: pRab10 is hypothesized to be primed by a phospho-ubiquitin code for E3 ligase-mediated proteasomal degradation, depleting functional Rab10. (2) Homeostatic window. LRRK2 kinase activity operates within a narrow physiological range. Both hyperactivation (R1441G, G2019S) and loss-of-function impair autophagic flux. (3) Kinase deficiency. LRRK2 loss-of-function compromises αGDI-mediated Rab renewal and recycling. This depletes the Rab network and disrupts the Rab7 cycle at the multivesicular body (MVB)–lysosome interface. TBC1D15 drives Rab7 GTP hydrolysis, and this reaction strips Rab7 from MVBs. Once Rab7 is gone, Arl8b takes over, and autophagosome-lysosome fusion proceeds. When the handover from Rab7 to Arl8b fails, fusion stalls. Convergence: both hyperactivation and deficiency converge on ALP stasis and a proteolytic bottleneck, leading to accumulation of protein aggregates (Aβ42, α-synuclein, mutant huntingtin [mHtt]) and autophagic substrates (e.g., p62)

The discovery of the LRRK2-Rab axis has substantially reshaped the molecular understanding of neurodegeneration. LRRK2 is a large, multidomain protein that contains both a ROC-COR (Ras of complex proteins-C-terminal of ROC) GTPase domain and a kinase domain, and this architecture couples genetic susceptibility to trafficking failure. Through phosphorylation of a defined subset of Rab GTPases, LRRK2 determines the fate of intracellular cargoes. Hyperactivation of LRRK2 causes trafficking stasis, which is increasingly recognized as a key contributor to AD, PD, and HD (Fig. 2).

### The LRRK2-Rab phosphorylation axis: from physiological homeostasis to the pathological driver

Under physiological conditions, LRRK2 acts as a feedback-controlled regulator of lysosomal homeostasis. When lysosomes are stressed, the kinase is recruited to the organelle and phosphorylates Rab10 at Thr73, thereby adjusting lysosome size, regeneration, and positioning for efficient cargo degradation [57, 58]. LRRK2 also plays a parallel role in immune surveillance. It is recruited to phagosomes that contain pathogens and again acts on Rab10. This role is context-dependent. LRRK2 can promote xenophagy under certain conditions, yet during *Mycobacterium tuberculosis* infection it hinders phagosome maturation [18]. Together, these findings indicate that homeostatic kinase activity must remain within a narrow window. Hyperactivation, as seen in carriers of the R1441G mutation, disrupts transport. Complete kinase deficiency likewise impairs autophagic flux and causes lysosomal enlargement with sequestosome-1 (p62) accumulation reminiscent of pathogenic overexpression [18, 59].

When this homeostatic regulation fails, LRRK2 acts as a central hub of the endolysosomal and autophagy-lysosomal pathways. It directly targets a subset of Rab GTPases on a conserved switch-II residue, including Rab8A, Rab10, and Rab12 [60]. Subsequent proteomic analysis expanded the substrate list. Rab3A/B/C/D, Rab8B, Rab35, and Rab43 are endogenous substrates, and Rab29 is additionally phosphorylated in overexpression systems [61]. These substrates span secretory, endocytic, and autophagic routes. Rab29 also functions as an upstream activator that recruits LRRK2 to the trans-Golgi network [8]. More recent work identifies Rab5 as a pathological substrate upon α-synuclein-induced LRRK2 activation [12]. Phosphorylated Rab12 (pS106-Rab12) is upregulated in AD and Lewy body disease. It localizes to granulovacuolar degeneration bodies during early tau deposition. It has therefore been proposed as an early biomarker of tau pathology, distinct from the later accumulation of pT73-Rab10 [10].

Rab10 is the most thoroughly characterized substrate. Pathogenic mutations in the ROC-COR GTPase domain of LRRK2 (such as the R1441G variant) can lead to trafficking dysfunction. The R1441G variant suppresses the intrinsic GTPase activity of LRRK2 and promotes GTP binding, raising LRRK2 kinase activity by three to four folds [57, 60, 62, 63]. This constitutive enzymatic hyperactivity reshapes the Rab phosphorylation profile. In freshly isolated peripheral blood neutrophils from R1441G carriers, Rab10-Thr73 phosphorylation is increased approximately 4.5-fold regardless of clinical disease status [57]. This finding indicates a cell-autonomous biochemical phenotype rather than a marker of degeneration severity [57, 64]. Compared with peripheral blood mononuclear cells, which show substantial inter-individual variability in pRab10 quantification, neutrophils provide a more reliable readout. With baseline adjustment, pRab10 measured in neutrophils can serve as a robust pharmacodynamic indicator of LRRK2 kinase inhibition [57, 64]. By contrast, the kinase-domain mutation G2019S elevates activity more modestly, by roughly two-fold, yet still remodels the Rab phosphorylation landscape over time [57, 63].

The dose-dependent relationship between LRRK2 kinase output and Rab network disruption is steep for R1441G and gradual for G2019S. This suggests that Rabopathy can arise from both acute dramatic enzymatic catastrophe and chronic moderate stress. Most LRRK2-Rab phosphorylation data come from cellular and biochemical assays. The stoichiometry of endogenous Rab phosphorylation should be confirmed in primary human neurons.

This vulnerability is bidirectional. Aged *Lrrk2*^−/−^ knockout mice develop lysosomal enlargement, impaired autophagy, accumulation of α-synuclein aggregates, and apoptotic cell death [65]. In an acute hepatic encephalopathy model, LRRK2 deficiency exacerbates lysosomal membrane permeabilization and neuronal damage. Complete kinase silencing can therefore itself be deleterious [66]. The following section discusses the mechanistic implications of this phenotypic convergence.

### Amplification of the LRRK2-Rab phosphorylation axis: a hypothetical phospho-ubiquitin code for Rab degradation

The LRRK2-Rab phosphorylation axis operates through feedforward mechanisms. Transient phosphorylation is converted into a durable loss of trafficking capacity. In parallel, Rab5 hyperactivation enlarges early endosomes [67]. This enlargement prolongs the residence of APP in an acidic environment with β-site APP-cleaving enzyme 1 (BACE1) and promotes Aβ42 generation [67]. Whether Aβ42 directly reactivates LRRK2 in return remains speculative. Current evidence supports an indirect feedback loop. LRRK2 hyperactivation drives endosomal APP trapping and Aβ42 production. Aβ42-induced cellular stress (oxidative stress and lysosomal membrane permeabilization) may perturb LRRK2 kinase homeostasis (Fig. 2). This cascade is particularly damaging to dopaminergic neurons. LRRK2-induced phosphorylation of Rab10 prevents its binding to Rab GDI factors necessary for membrane delivery and recycling. This interferes with dopamine vesicle recycling and promotes α-synuclein accumulation [60, 68–70]. Autophagic degradation falters in parallel. Microtubule-associated protein 1 light chain 3 beta-II (LC3B-II) and p62 accumulate, and pro-cathepsin D is not processed into its mature, active form within lysosomes [66, 71].

Both LRRK2 hyperactivation and LRRK2 deficiency disrupt autophagic flux and produce similar lysosomal abnormalities [65, 71, 72]. If excessive phosphorylation were merely a reversible signaling event, it would be hard to explain why kinase overactivity phenocopies kinase loss. In many signaling pathways, phosphorylation serves not only to modulate function but also to mark proteins for degradation. Phosphorylation creates docking sites for E3 ubiquitin ligases, and in doing so it links kinase activity to proteasomal clearance. We therefore hypothesize that LRRK2-tagged Rab GTPases are earmarked for E3 ligase recruitment, followed by ubiquitination and proteasomal degradation. A transient signal would be converted, step by step, into the durable disappearance of trafficking capacity. As functional Rabs dwindle, neurons can no longer sustain autophagosome-lysosome fusion or endosomal cargo sorting. Notably, this framework accounts for the phenotypic convergence between hyperactivation and knockout. Both perturbations compromise autophagic flux because both converge on the same final state, a depleted Rab transport network, driven by excessive degradation (hyperactivation) in one case and by insufficient renewal (deficiency) in the other.

However, knowledge gap remains in this model: the E3 ligases that recognize LRRK2-phosphorylated Rabs are unknown, and the relevant ubiquitination sites are also unclear. The model rests on two observations: (1) LRRK2 phosphorylation of Rabs disrupts their interactions with canonical effectors and GDIs [60], and (2) LRRK2 gain and loss result in similar defects in autophagic flux [65,71,72]. These findings may suggest the following sequence of events: Rab phosphorylation first disrupts Rab-effector/GDI interactions, and sustained dysregulation may reduce functional Rab capacity, thereby impairing endolysosomal and autophagic transport and gradually progressing to the trafficking stasis that we define as neurodegenerative Rabopathy.

## Cross-disease convergence: the ALP as a shared endophenotype of Rabopathy

ALP is the common destination at which misfolded proteins accumulate across diseases. Rab7 is central to this convergence. Together with soluble N-ethylmaleimide-sensitive factor attachment protein receptor (SNARE) complexes, Rab7 regulates autophagosome-lysosome fusion. In healthy neurons, Rab7 localizes predominantly to late endosomal and lysosomal membranes rather than early endosomes, and the mechanisms underlying its recruitment to autophagic structures remain partially defined. Rab7 function is modulated by RAS oncogene family-like 1 (*RAB7L1*; one of five genes in the PD risk-associated *PARK16* locus; also known as Rab29, distinct from the late endosomal Rab7), which interacts with *LRRK2* to determine PD risk [73, 74].

An ordered molecular handover controls this lysosomal gateway. TBC1 domain family member 15 (TBC1D15), a GTPase-activating protein (GAP), deactivates Rab7 and removes it from mature multivesicular bodies (MVBs) [75]. The ADP-ribosylation factor-like 8b (Arl8b)/SifA and kinesin-interacting protein (SKIP) complex participates in this regulated disengagement. Impaired Rab7 cycling—whether through constitutively active Rab7 mutants that cannot complete GTP hydrolysis, disrupted Arl8b recruitment, or other handover defects—traps autophagic cargoes in immature compartments and prevents their delivery to the proteolytic environment.

Proteostasis depends on the molecular handover. Rab7 must yield to Arl8b. Every intracellular aggregate, from α-synuclein to mutant huntingtin to Aβ, reaches the lysosome through this route. This step may be especially vulnerable in PD. The *PARK16* locus provides the genetic evidence. In Ashkenazi Jewish populations, the protective rs1572931 minor allele limits exon skipping in *RAB7L1* mRNA. A robust Rab29-dependent sorting network is maintained at the Golgi and endolysosomes, reducing the PD risk [73, 74, 76]. The mechanism that links Rab29 failure to Rab7-mediated fusion remains unclear. Large GWAS meta-analyses have reinforced this link. They independently confirm *PARK16* as a susceptibility locus [77].

Early endosomal dysfunction appears before neurons die in AD. Enlarged Rab5-positive early endosomes are found in postmortem human brains and in transgenic mouse models. These swollen compartments impede traffic. The backlog then burdens the Rab7-dependent degradative pathway [67, 78]. Postmortem studies provide further support. In vulnerable regions such as the basal forebrain, frontal cortex, and hippocampus, both Rab5 effectors and the late endosomal component Rab7 are upregulated [79]. A plausible reading is that neurons mount a compensatory response to the rising degradative demand. Whether the extra Rab7 is actually cycling, or merely parked in a stalled pool, is still unknown. Genetic validation of the role of Rab7 in AD neurodegeneration is likewise pending. Either way, the endpoint is the same. Autophagosome-lysosome fusion loses efficiency, and autophagic substrates pile up.

Autophagosomes mature on schedule in HD, but they cannot fuse with lysosomes. The culprit is mutant huntingtin, which disrupts the miR-1-TBC1D15-Rab7 axis [80]. Evidence for this axis came from studies in cells and flies. Whether it operates the same way in human patients remains an open question, one that only patient-derived systems can answer.

**Fig. 3:**
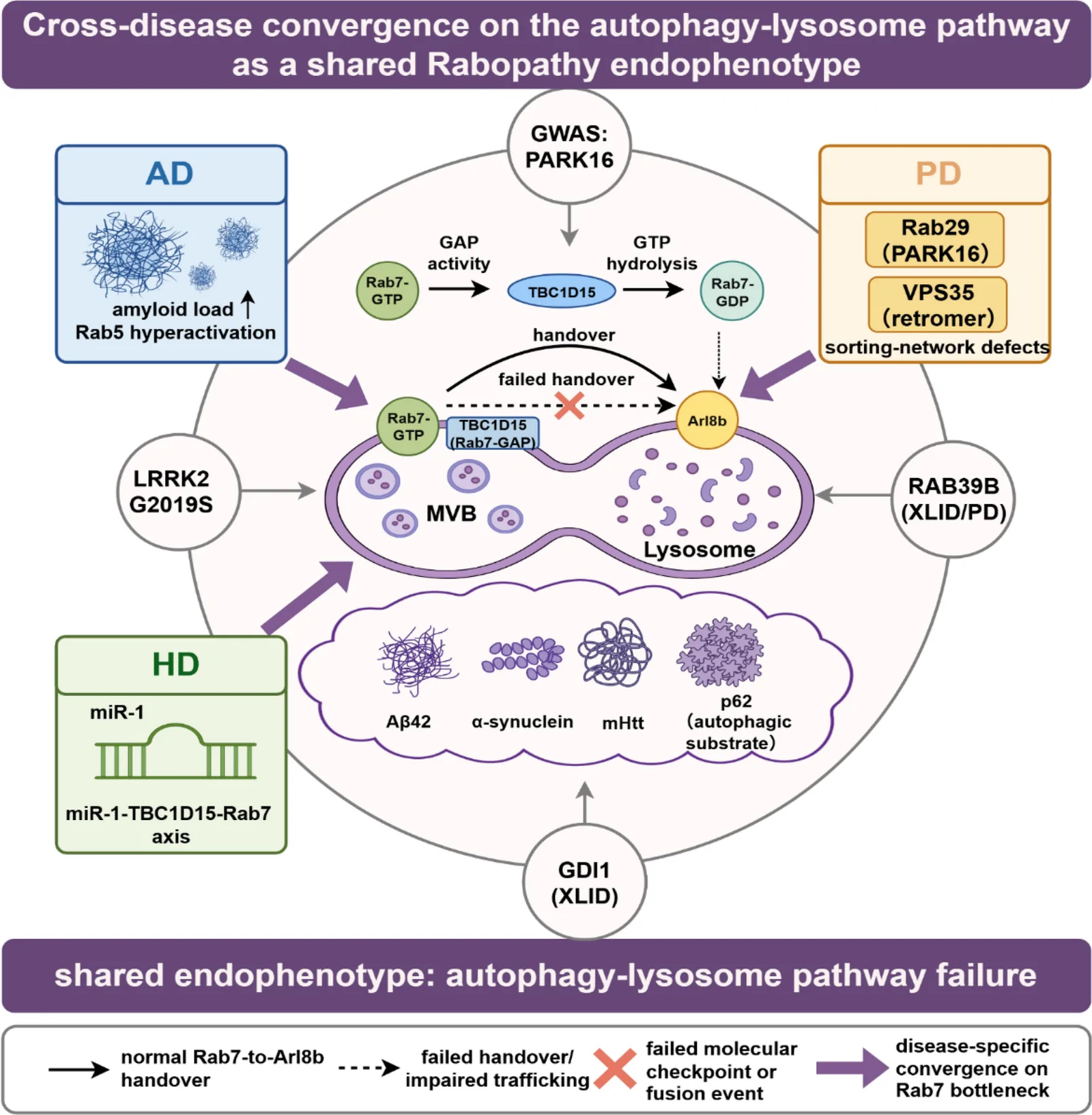
Cross-disease convergence on the autophagy-lysosome pathway as a shared Rabopathy endophenotype. The central panel shows how Rab7 hands over to Arl8b in a stepwise fashion at the MVB-lysosome interface.TBC1D15-mediated Rab7 GTP hydrolysis enables removal of Rab7-GDP from MVBs, permitting Arl8b engagement and autophagosome-lysosome fusion (solid arrows). When this handover fails (dashed arrow with red cross), ALP stasis occurs. AD, PD, and HD each disrupt this pathway at distinct steps. AD involves amyloid load and Rab5 hyperactivation. PD involves Rab29/*PARK16* and VPS35/retromer sorting defects. HD involves the miR-1-TBC1D15-Rab7 axis. Human genetic evidence from GWAS (*PARK16*) and causal mutations (*LRRK2* G2019S, *RAB39B*, *GDI1*), further links this pathway to neurodegeneration. Failure of the Rab7-to-Arl8b handover creates a shared substrate pool of undegraded protein aggregates (Aβ42, α-synuclein, mHtt) and accumulated autophagic substrates (e.g., p62)

*PARK16* risk variants (Rab29) converge on Rab7-mediated sorting [73, 74]. These findings support the notion that Rabopathy represents a convergent pathogenic mechanism across neurodegenerative diseases (Fig. 3; Table 1). Enhancing the Rab7 cycle through modulation of GAPs such as TBC1D15 could improve lysosome maturation and degradation in multiple proteinopathies. This would mark a conceptual shift from disease-specific targets toward restoration of the shared degradative pathway, though the Rab7-directed interventions remain at the preclinical stage.

**Table 1:** The Rabopathy spectrum: Rab GTPases and their regulators in nervous-system disorders

Rab/Regulator	Subcellular localization and function	Associated disorder(s)	Pathogenic mechanism	Evidence level
Rab3A (*RAB3A*)	Synaptic vesicle reserve pool and docking	AD, HD, intellectual disability	Reserve-pool depletion; Rab3A–rabphilin-3A docking failure	Mechanistic + biomarker
Rab27B	Recruitment into the readily releasable pool	Age-related cognitive decline	Defective GDI-mediated recycling and phosphorylation impairs rapid sequential processing	Mechanistic
Rab4	Fast recycling endosome	Stress-related cognitive impairment	SGK1–αGDI (Ser213) control of AMPAR recycling	Mechanistic
Rab5	Early endosome	AD, Down syndrome	Increased specific activity (GEF/GAP imbalance) drives endosomal enlargement and APP–BACE1 encounter	Mechanistic
Rab7	Late endosome/MVB	AD, HD	Failure of TBC1D15-mediated Rab7-to-Arl8b handover blocks autophagosome–lysosome fusion	Mechanistic
Rab29 (*RAB7L1, PARK16*)	trans-Golgi network/retromer	PD	GWAS risk locus; sorting-network defects converging on Rab7	Human genetic association
Rab8A	Post-Golgi transport and cilia	PD, HD	Sequestration by pS129-α-synuclein; possible impairment of DAT surface delivery (unresolved); interrupted lysosomal supply	Mechanistic
Rab10	ER–Golgi and lysosomal homeostasis	PD	LRRK2 substrate (pThr73); pRab10 disrupts ciliogenesis and lysosomal function	Human genetics + biomarker
Rab11	Recycling endosome	HD	mHtt disrupts effector interactions, sequesters HIP1 and impairs actin-based transport, reducing receptor recycling	Mechanistic + therapeutic rescue
Rab12	Autophagosome/lysosome, autophagic flux regulation	PD	LRRK2 substrate (pSer106); defective autophagic flux	Mechanistic
Rab35	Endosomal/exosomal release	PD	Aberrant α-synuclein secretion and spread; elevated serum Rab35 distinguishes PD from MSA	Biomarker + mechanistic
RAB39B	ER–Golgi checkpoint	XLID, early-onset PD	Loss of function causes GluA2-AMPAR retention and α-synuclein dyshomeostasis; reversible phenotype	Human causal genetics + mechanistic
αGDI (*GDI1*)	Cytosolic Rab recycling	XLID	Interrupted Rab recycling compromises neuronal and glial trafficking	Human genetics
DENND5B (GEF)	Golgi/cytosol, GEF for RAB39A/B	Neurodevelopmental disorders	De novo mutations cause cognitive impairment, epilepsy and white-matter defects	Human genetics
TBC1D15 (GAP)	MVB/lysosome, GAP for Rab7	HD	miR-1-TBC1D15-Rab7 axis imbalance	Mechanistic
LRRK2 (upstream kinase)	Cytosol/lysosome/Golgi, a master kinase of Rab phosphorylation	PD (AD-linked)	G2019S/R1441G increase phosphorylation of selected Rabs in a mutation- and substrate-specific manner	Human causal genetics

Evidence level: human causal genetics > human genetic association > biomarker > mechanistic (animal/cell). AD, Alzheimer's disease; HD, Huntington's disease; PD, Parkinson's disease; XLID, X-linked intellectual disability; MSA, multiple system atrophy; MVB, multivesicular body; GWAS, genome-wide association study; DAT, dopamine transporter; GEF, guanine nucleotide exchange factor; GAP, GTPase-activating protein

## Disease-specific dysregulation of the Rab network

**Fig. 4:**
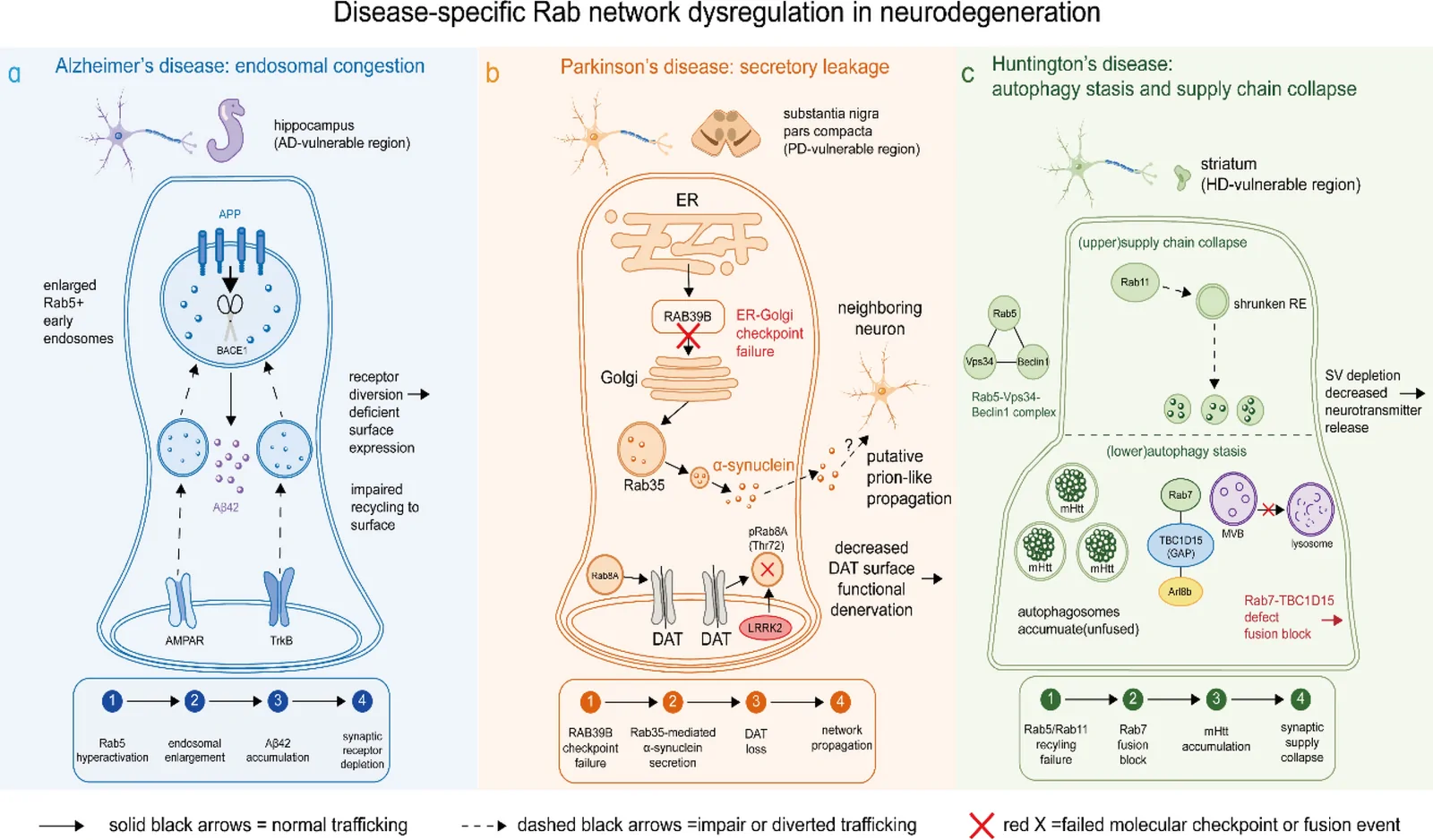
Disease-specific Rab network dysregulation in neurodegeneration. **a** Alzheimer's disease. In the hippocampus, Rab5 hyperactivation leads to enlargement of early endosomes. This traps APP with BACE1 and promotes Aβ42 generation. AMPARs and TrkB are diverted away from the synaptic membrane. **b** Parkinson's disease. In the substantia nigra, three defects converge. RAB39B checkpoint failure at the ER-Golgi interface disrupts vesicle sorting. Rab35 drives α-synuclein secretion. LRRK2 phosphorylates Rab8A at Thr72 and may reduce dopamine transporter (DAT) surface expression, a link that remains unresolved. The net result is functional denervation. **c** Huntington's disease. In the striatum, Rab11-dependent recycling endosome failure depletes synaptic vesicles (upper). Rab7-TBC1D15 dysfunction blocks autophagosome-lysosome fusion and causes mutant huntingtin accumulation (lower).

Although the LRRK2-Rab axis and ALP are implicated in multiple disorders, the clinical heterogeneity of neurodegenerative diseases arises from the selective failure of distinct Rab sub-networks. Below, we examine how AD, PD, and HD represent distinct Rabopathies with characteristic molecular signatures (Fig. 4).

### AD: Rab5-driven endosomal congestion and synaptic receptor depletion

AD is increasingly seen as a disease that begins with intracellular trafficking failure. Extracellular plaques may not be the whole story. In the postmortem frontal cortex of AD patients, Rab5-positive early endosomes are abnormally enlarged [78, 81]. This phenotype appears early in the disease course. It may be an initiating event rather than a passive downstream consequence. Recent biochemical work has clarified the molecular basis of this enlargement. Earlier studies found elevated Rab5 protein levels in the hippocampus and frontal cortex [78, 79]. A newer analysis of postmortem tissue from Down syndrome patients points to a different cause. Rab5 hyperactivation arises from an imbalance between GEFs (guanine nucleotide exchange factors, which promote the exchange of GDP for GTP) and GAPs (GTPase-activating proteins, which catalyze GTP hydrolysis). When this balance is disturbed, Rab5 stays longer in its GTP-bound active state [82]. Early endosomal membrane dynamics are then perturbed and the compartment physically expands [83]. The enlarged endosome creates an acidic microenvironment. APP remains inside for longer time and is processed more extensively by β-site APP-cleaving enzyme 1 (BACE1). Aβ42 production rises as a result [67, 78, 84]. Endosomal dysfunction in AD is thus a defect of Rab5 cycling kinetics. The timing of Rab5 activation and inactivation no longer matches the physiological demand.

Sustained endosomal processing of APP, however, imposes a competitive cost on synaptic maintenance. Neuronal membrane trafficking operates under finite capacity constraints. As the endosomal machinery processes increasing Aβ burdens, transport resources are diverted from synaptic renewal. Direct measurements of trafficking flux diversion in individual neurons will require compartment-specific biosensors. Consistent with this model, Rab5 overexpression suppresses tropomyosin receptor kinase B (TrkB) expression in cells, whereas TrkB knockdown does not alter Rab5 levels, placing endosomal dysfunction upstream of neurotrophic starvation [78]. Hippocampal Rab5 gene expression levels correlate negatively with antemortem cognitive scores (Mini-Mental State Examination [MMSE] and Global Cognitive Score [GCS]; *P* < 0.001) [78]. Synaptic protein depletion in the frontoparietal cortex of AD cases reflects exhaustion of the synaptic vesicle reserve pool as trafficking capacity is redirected toward endosomal cargo processing [85]. We propose that cognitive impairment in AD arises, at least in part, from synaptic resource diversion. The neuronal trafficking network, overwhelmed by the demand to clear misfolded proteins, can no longer sustain the membrane dynamics required for memory formation.

Accordingly, therapeutic strategy should shift from simply clearing Aβ toward restoring trafficking equilibrium. In Down syndrome neuronal models, endosomal enlargement and degenerative phenotypes can be rescued by genetic suppression of Rab5 [86]. Whether this strategy proves useful in sporadic AD remains to be tested across diverse genetic backgrounds. An alternative approach is to suppress adaptor protein complex 2 subunit sigma 1 (AP2S1). In cellular models and APP/PS1 mice, AP2S1 silencing promotes the translocation of APP from RAB9-positive late endosomes to LAMP1-positive lysosomes. It also enhances late endosome–lysosome fusion. APP and Aβ levels decrease as a result, and cognitive performance improves [87].

### PD: secretory leakage and functional denervation

Unlike Rabopathy in AD, the intracellular trafficking defects are converted into extracellular pathological spread in PD. The dopaminergic neurons of the substantia nigra are particularly vulnerable to secretory failure, largely due to their high metabolic and trafficking demands.

At the endoplasmic reticulum (ER)–Golgi interface, RAB39B functions as a molecular checkpoint for proper cargo sorting and vesicle budding. Dysfunction of this checkpoint results from pathogenic *RAB39B* mutations. The p.Trp186stop nonsense variant and the p.G192R mislocalization variant are two examples. ER-to-Golgi trafficking is then impaired. α-Synuclein homeostasis is dysregulated as a result. This creates an aggregation-prone condition that seeds early-onset PD [14, 88–90].

A second defect involves Rab35, a protein with dual roles in PD pathogenesis. In healthy neurons, Rab35 supports neurite outgrowth [35]. However, the level of Rab35 elevates markedly in the substantia nigra across PD models (1-methyl-4-phenyl-1,2,3,6-tetrahydropyridine, rotenone, and LRRK2-G2019S transgenic models) and in the serum of PD patients [41]. Cross-sectional clinical data link increased serum Rab35 to earlier onset of PD, by a median of five years, and to longer disease duration. Whether it can serve as a prodromal marker needs to be validated in longitudinal studies [41, 90]. Rab35 overexpression promotes the aggregation and secretion of α-synuclein [41], and LRRK2-mediated phosphorylation of Rab35 enhances the intercellular propagation of α-synuclein aggregates, partly by attenuating their lysosomal degradation [37]. Rab35 is an established regulator of exosome release, with its inhibition leading to intracellular accumulation of endosomal vesicles and impairment of exosome secretion [36]. Disruption of the endosomal sorting complex required for transport III (ESCRT-III) machinery likewise increases cellular release of α-synuclein [91]. It is therefore plausible that the Rab35-dependent vesicular secretion turns cell-autonomous α-synuclein accumulation into prion-like, circuit-wide spread [90].

Rab8A dysfunction adds a third layer of defect, by leading to functional denervation at the presynaptic membrane. Rab8A regulates post-Golgi trafficking and, once recruited to stressed lysosomes in an LRRK2-dependent manner, maintains lysosomal homeostasis [58]. Two converging insults compromise this pathway in PD. First, α-synuclein physically interacts with the switch region of Rab8A in a Ser129 phosphorylation-dependent manner [92]. Yin et al. originally interpreted this interaction as adaptive, because Rab8A binding enhances α-synuclein aggregation yet reduces its cellular toxicity [92]. The switch region, however, governs GTPase activity and effector binding, so persistent engagement by phosphorylated α-synuclein may sequester Rab8A away from functional trafficking. This is supported by evidence from biochemical and cell-based assays and further validation in human dopaminergic neurons is needed. Second, LRRK2-mediated phosphorylation of Rab8A at Thr72 disrupts its interaction with effector proteins [8, 60]. These insults compromise Rab8A-dependent post-Golgi delivery and lysosomal stress resilience at the presynaptic terminal. Whether this extends to defined cargoes such as the dopamine transporter, and the resultant impaired dopamine clearance, remains an open question.

In addition, the Rab28/Park7 (DJ-1) pathway provides further insights into the PD-associated Rabopathy. In rab28-deficient zebrafish retina, the redox sensor Park7 (DJ-1), a product of a familial PD gene, is dysregulated selectively at the dawn and the dusk peaks of outer-segment phagocytosis, two time points of maximal membrane turnover in the retinal pigment epithelium [93]. The observation comes from retina rather than dopaminergic neurons, but it suggests that Rab proteins may buffer redox homeostasis during high-frequency membrane renewal; failure of this function could be especially consequential in metabolically demanding neurons [93].

In summary, while AD Rabopathy produces internal congestion, PD Rabopathy is characterized by impaired secretory fidelity: dysregulation of Rab35, RAB39B, and Rab8A disrupts the sorting decisions that determine intracellular retention versus extracellular release. α-Synuclein is aberrantly secreted, leading to circuit-level propagation. Meanwhile, impairment of Rab8A-dependent presynaptic maintenance produces a functional leakage of dopaminergic signaling integrity.

Therefore, PD therapeutic development might be reoriented from anti-aggregation toward anti-secretion strategies. Diagnostically, serum Rab35 can distinguish PD from multiple system atrophy (MSA), yielding a receiver operating characteristic area under the curve of 0.79, which rises to 0.91 when combined with serum α-synuclein [94]. This distinction is clinically meaningful, since MSA, as a separate synucleinopathy, exhibits distinct Rab-mediated secretory profiles [94]. At the presynaptic level, dopaminergic terminals may be protected from functional denervation by preserving Rab8A-mediated post-Golgi trafficking together with lysosomal resilience; at the secretory level, inhibition of Rab35 or stabilization of RAB39B may restore the integrity of the secretory pathway and limit α-synuclein spread.

### HD: disruption of ALP and the synaptic supply chain

As an autosomal-dominant neurodegenerative disorder, HD is caused by cytosine-adenine-guanine (CAG) trinucleotide repeat expansion in the huntingtin (*HTT*) gene. In HD, Rab dysfunction spans multiple trafficking levels, ranging from recycling endosomes and autophagic degradation to the Golgi-derived supply chain.

Early autophagic initiation is compromised by Rab5 dysfunction. Rab5 recruits the endosomal Vps34 complex to generate phosphatidylinositol 3-phosphate (PI3P) on endosomes [95]. Rab5-mediated macroautophagy modulates mutant huntingtin aggregation and toxicity in cellular and *Drosophila* HD models [96]. Rab5 impairment is thus an upstream defect. It places excessive burden on the downstream Rab11 recycling and Rab7 degradative systems.

The next pathological cascade is the collapse of the Rab11-mediated synaptic supply chain. Mutant huntingtin interferes with Rab11-effector interactions required for vesicle formation from recycling endosomes. It sequesters Rab11-interacting proteins such as huntingtin-interacting protein 1 (HIP1) and disrupts the actin dynamics on which the transport of Rab11-positive recycling endosomes depends [97]. Defective Rab11-dependent vesicle formation has been observed in HD patient fibroblasts and knock-in mouse models [97]. It has also been observed in primary neurons from HD mice with glucose hypometabolism [98]. In a *Drosophila* model of HD, expression of mutant huntingtin decreases presynaptic vesicle size through impaired Rab11 activity and altered HIP1 interaction, and reduces synaptic transmission [99]. Restoring Rab11 expression normalizes vesicle size, synaptic transmission, and locomotor behavior, which identifies Rab11 activation as a candidate therapeutic strategy [99]. However, the therapeutic window for Rab11 activation in HD remains undefined. Excessive Rab11 activity may disrupt the narrow physiological balance required for trafficking homeostasis, as shown by the dosage sensitivity of RAB39B described above.

Defects of the Rab3A-rabphilin-3A docking complex add a presynaptic dimension. Rabphilin-3A is an effector supporting vesicle docking and release [100]. In the HD sensory-motor cortex, rabphilin-3A is depleted together with the SNARE protein synaptosome-associated protein 25 (SNAP-25), whereas Rab3A and synaptophysin remain unchanged, indicating selective impairment of the docking complex rather than generalized synaptic loss [101]. Preserving Rab3A-mediated vesicle priming, or stabilizing rabphilin-3A, may therefore offer an additional presynaptic therapeutic target complementary to Rab11 rescue.

Rab7 dysfunction further creates an autophagic bottleneck. Rab11 governs membrane recycling, whereas Rab7 executes the terminal clearance of protein aggregates. Rab7 mediates the fusion of autophagosomes with lysosomes, the final proteolytic step of the ALP [75]. In HD, dysregulation of the miR-1-TBC1D15-Rab7 axis may impair autophagic flux. Stress elevates the Rab7 GAP, TBC1D15. Excessive Rab7-GTP hydrolysis follows. Autophagosome-lysosome fusion is then reduced. Autophagosomes pile up in a mature but undocked state. They are loaded with mutant huntingtin yet cannot deliver their cargo. This axis is a potentially druggable regulatory node. Interferon-β (IFN-β) can act on this node. It induces expression of miR-1, which then represses TBC1D15 translation [80]. The reduction of TBC1D15 lowers Rab7 activity and thereby stimulates macroautophagy [80]. This improves clearance of mutant huntingtin. Systemic IFN-β administration remains problematic in practice, however. The blood–brain barrier limits its CNS delivery. Systemic immunomodulatory effects are another concern.

Rab8 disruption breaks the Golgi-to-lysosome supply chain as well. In healthy neurons, Rab8 drives post-Golgi transport of cathepsin-containing vesicles and lysosomal membrane proteins that maintain degradative capacity. Mutant huntingtin delocalizes the Rab8/optineurin complex from the Golgi apparatus and diverts vesicle traffic away from lysosomal destinations [102]. The neuronal trafficking machinery is thereby redirected toward managing the growing aggregates, at the expense of metabolic maintenance, so Rab8 dysfunction becomes a rate-limiting defect in HD pathogenesis.

In summary, these findings suggest a shared therapeutic logic: restoration of endolysosomal flux. Synaptic vesicle size and behavior can be normalized by Rab11 rescue, the autophagic bottleneck imposed by mutant huntingtin could be relieved through enhanced Rab7 cycling or Rab8-mediated lysosomal supply, and presynaptic release competence may be restored by preserving the Rab3A-rabphilin-3A docking complex [99, 103]. Synaptic dysfunction is an early, reversible feature of HD that precedes neuronal death, so interventions aimed at this stage of Rabopathy are the most likely to modify disease course.

## Therapeutic perspectives within the Rabopathy framework

### The Rab35–ESCRT pathway: activity-dependent degradation and synaptic quality control

The Rab35**–**ESCRT pathway acts as a quality control and degradation route for ubiquitinated synaptic cargo, limiting the accumulation of potentially toxic material. This pathway is regulated by Skywalker (Sky), a neuronal GTPase-activating protein for Rab35 that governs synaptic vesicle endosomal sorting [104].

In *Drosophila* *sky* mutants, the readily releasable synaptic vesicle pool is significantly enlarged (3142 ± 238 quanta versus 2108 ± 188 in controls), and its refilling rate doubles the control (8239 ± 1269 quanta/s) [104]. Removing a single copy of ESCRT component genes (*hrs, vps23, vps25*, or *vps32*) suppresses this enhancement, which indicates that Rab35 and ESCRT cooperate to clear ubiquitinated synaptic proteins such as ubiquitinated neuronal synaptobrevin-HA [104].

The Rab35–Sky–ESCRT axis functions as an activity-dependent quality control system that is mechanistically distinct from developmental pruning and that eliminates damaged or excess synaptic proteins after release. Expression of dominant-negative Rab35 (Rab35^DN^) restores normal quantal content in *sky* mutants, positioning Rab35 as a high-precision regulator of synaptic strength [104]. Beyond its action on synaptic vesicle proteins, Rab35 also promotes the ESCRT-dependent endolysosomal degradation of tau, coupling vesicle recycling to proteostasis [105]. These dual functions make Rab35-GAP enhancers (Sky mimetics) an attractive therapeutic concept: rather than broadly inhibiting LRRK2, they would act selectively on synaptic quality control and avoid systemic kinase-related side effects. This axis is conserved from *Drosophila* to mammals, but the specific pharmacological properties of Sky mimetics, including blood–brain barrier penetrance and selectivity for neuronal Rab35 pools, have yet to be characterized.

### Post-translational modifications (PTMs) beyond LRRK2 phosphorylation

In addition to LRRK2, stress-responsive PTMs determine whether trafficking adapts or fails. As discussed earlier, LRRK2-mediated phosphorylation may hypothetically prime Rabs for ubiquitin-dependent degradation (the phospho-ubiquitin code). Here we focus on better-characterized PTMs that regulate receptor trafficking directly.

Under acute stress, corticosterone-activated serum- and glucocorticoid-inducible kinase 1 (SGK1) phosphorylates αGDI at Ser213. This enhances GDI-Rab4 complex formation and Rab4-mediated AMPAR recycling, transiently raising synaptic transport capacity. The phosphomimetic GDI mutant S213D enhances AMPAR function, whereas the non-phosphorylatable S213A mutant reduces it. This demonstrates that GDI phosphorylation adjusts trafficking capacity in the brain under stress [34, 106]. Conversely, p38 mitogen-activated protein kinase (p38 MAPK) phosphorylates Rab5 and promotes clathrin-dependent AMPAR internalization during long-term depression [107, 108].

These two PTM branches form a regulatory balance. The SGK1-GDI-Rab4 axis supports synaptic strengthening. The p38-Rab5 signaling mediates synaptic weakening. During chronic neuroinflammation, persistent p38 activation may lock this switch in an internalization-dominant state and contribute to synaptic loss in AD. Cognitive decline may result from PTM-driven synaptic inflexibility. The dynamic balance between AMPAR insertion and internalization becomes fixed in a pathological equilibrium, either constitutively active (excitotoxicity) or inactive (synaptic silencing), precluding activity-dependent remodeling. By modulating discrete sites such as GDI-Ser213, flexibility may be restored without perturbing the entire Rab network. However, before the therapeutic potential of GDI-targeted interventions can be validated, the spatiotemporal dynamics of GDI-Ser213 phosphorylation in distinct brain regions and under chronic stress conditions still require further investigation.

### Technological advances enabling mechanistic precision

Progress in technologies such as super-resolution imaging, artificial intelligence (AI)-based structural prediction, and single-cell omics have shifted the field toward mapping the organization of Rabs and their partner proteins in situ. Super-resolution microscopy has resolved BIN1 localization and active-zone protein cluster organization at presynaptic sites [109]. AlphaFold-Multimer and related deep-learning methods predict the complex structures of LRRK2-Rab assemblies, informing the rational design of allosteric inhibitors. Proximity ligation assays now detect active LRRK2 and phosphorylated Rab10 in situ at cellular resolution. In postmortem tissues from idiopathic PD patients and in rat models, this approach revealed selective LRRK2 activation in dopaminergic neurons of the substantia nigra [9]. Correlative light and electron microscopy, combined with proximity-dependent biotin identification (BioID)-SKIP labeling, has provided ultrastructural validation of the ordered Rab7-to-Arl8b handover at the lysosomal gate [75]. CRISPR-engineered cell lines expressing endogenous CD63 and Arl8b have further freed these analyses from overexpression artifacts, allowing therapeutic targets to be grounded in physiological cell states [75]. However, these technologies remain limited by cost, technical expertise, and the inherent difficulty of detecting low-abundance Rab GTPases at single-cell resolution. Such spatial precision is therapeutically essential, because precise locating of the Rab(s) that have failed and specific interaction(s) that have been blocked is critical for effective intervention.

### Therapeutic targets and clinical translation

A spectrum of strategies aimed at restoring Rab network integrity has emerged. The first large-scale clinical test is the Phase 2b LUMA trial of the LRRK2 inhibitor BIIB122/DNL151 [110]. Robust peripheral target engagement (> 90% kinase inhibition) was achieved, yet disease progression in early-stage PD was not slowed [110]. A cerebrospinal fluid sub-study observed only an approximately 30% reduction of phosphorylated Rab10, indicating incomplete central target engagement [110]. Accordingly, development in idiopathic PD was discontinued, although the Phase 2a BEACON study in carriers of pathogenic *LRRK2* variants is still ongoing [110]. These results, together with the observation that both excessive and insufficient LRRK2 activity impair autophagic flux [7, 66, 71, 72], support calibrated inhibition rather than maximal kinase suppression.

Complementary options include Rab subtype-selective modulators. Rab7 agonists remain a preclinical concept. In HD models, Rab11 overexpression normalized synaptic vesicle size and ameliorated locomotor deficits in a *Drosophila* model [99]. Genetic suppression of Rab5 with antisense oligonucleotides restored endosomal Rab activity and mitigated degenerative phenotypes in a Down syndrome model of AD [86].

Rab proteins also hold diagnostic promise. Phosphorylated Rabs (e.g., pRab10-Thr73) quantified in peripheral blood neutrophils have already served as pharmacodynamic biomarkers in LRRK2 inhibitor trials [57, 64]. Cerebrospinal fluid-based Rab profiling is under development for the differential diagnosis of synucleinopathies, although validation in large cohorts is still pending.

We propose that the field should shift from broad inhibition toward calibrated modulation. The therapeutic goal is not to ablate LRRK2 but to restore physiological Rab flux. Future therapeutics will likely target defined trafficking nodes (Table 2), such as Rab11 agonists in HD, Rab35 inhibitors and RAB39B stabilizers in PD, Rab7 modulators for autophagic clearance, Rab8A-mediated dopamine transporter (DAT) delivery, and GDI stabilizers in stress-related cognitive impairment. Intervening at the reversible stage of synaptic dysfunction, before neuronal death occurs, offers the most plausible route to disease modification across the Rabopathy spectrum. Most of these Rab-targeted strategies, however, remain conceptual or at the earliest preclinical stage. Their clinical development will require substantial investment in blood–brain barrier penetration, subtype selectivity, and long-term safety profiling.

**Table 2:** Rab network: therapeutic targets, strategies and translational status

Target/node	Disease	Strategy	Modality	Representative agent/evidence	Stage	References
LRRK2 kinase activity	PD	Calibrated kinase inhibition	Small-molecule inhibitor	DNL151/BIIB122: Phase 2b LUMA trial negative in idiopathic PD (NCT05348785); Phase 2a BEACON ongoing in LRRK2-associated PD (NCT06602193)	Phase 2b/2a (PD)	[110]
LRRK2 expression	PD	RNA-mediated knockdown	Antisense oligonucleotide	BIIB094/ION859: Phase 1 completed (NCT03976349)	Phase 1 (PD)	[111]
pRab10 (Thr73)	PD	Pharmacodynamic monitoring	Peripheral blood biomarker	Companion test in LRRK2 inhibitor trials	Phase 2 (PD)	[57, 64]
Rab5	AD/DS	Rab5 suppression	ASO-mediated knockdown	Rab5 suppression rescues degeneration in Down syndrome neuronal models	Preclinical (AD/DS)	[86]
Rab11	HD	Rab11 rescue	Gene replacement/overexpression	Rab11 rescue normalizes synaptic vesicle size and behavior in HD models	Preclinical (HD)	[99]
Rab3A–rabphilin-3A	HD	Rabphilin-3A stabilization	Small-molecule stabilizer (concept)	Depletion of rabphilin-3A observed in HD patient cortex (mechanistic observation)	Preclinical (HD)	[101]
Rab35–Sky–ESCRT	PD	Rab35-GAP/Sky activation	Small-molecule activator (concept)	In *Drosophila*, loss of the Rab35-GAP Sky/TBC1D24 facilitates endosomal trafficking of synaptic vesicles by controlling Rab35 GTPase activity; *ESCRT–sky* genetic interactions support replacement of dysfunctional synaptic vesicle proteins.	Preclinical (PD)	[104]
Rab7–TBC1D15	HD	Rab7/TBC1D15 modulation	miR-1 mimetic or TBC1D15 inhibitor (concept)	IFN-β–miR-1 represses TBC1D15, reduces Rab7 activity and stimulates autophagy (evidence from PD-like models)	Preclinical (HD)	[80]
RAB39B	XLID/PD	RAB39B replacement	Gene replacement (concept)	Reversible phenotype in mouse models	Preclinical (XLID/PD)	[49, 54]
αGDI/*GDI1* (Ser213)	Stress	GDI stabilizer or SGK1 modulator	Small-molecule modulator (concept)	Phosphomimetic S213D-GDI enhances AMPAR function	Preclinical (Stress)	[34, 106]
Serum Rab35	PD/MSA	Diagnostic biomarker	Serum immunoassay	Rab35 + α-synuclein differentiate PD from MSA (AUC 0.91)	Biomarker validation	[41, 94]

Stage definitions: Phase 1/Phase 2, clinical trial phase, including 2a/2b substudies where specified; Preclinical, in vitro or animal proof-of-concept; Biomarker validation, assay evaluated in clinical samples for diagnostic performance. AD, Alzheimer’s disease; AAV, adeno-associated virus; AMPAR, α-amino-3-hydroxy-5-methyl-4-isoxazolepropionic acid receptor; ASO, antisense oligonucleotide; AUC, area under the curve; CSF, cerebrospinal fluid; DAT, dopamine transporter; DS, Down syndrome; HD, Huntington’s disease; LRRK2, leucine-rich repeat kinase 2; MSA, multiple system atrophy; PD, Parkinson’s disease; XLID, X-linked intellectual disability

## Limitations of the Rabopathy framework

The Rabopathy framework carries several empirical and conceptual constraints. Much of the mechanistic evidence comes from cellular and animal models. Direct validation in human postmortem tissue or patient-derived neurons remains incomplete. Basic parameters are also unsettled. The stoichiometry of endogenous Rab phosphorylation in native human neurons, for instance, has yet to be established. The evidence base varies considerably across diseases. The LRRK2-Rab phosphorylation axis is well characterized in PD. Yet its proposed role as a shared amplifying mechanism in AD and HD rests on indirect or preliminary evidence. Moreover, each condition may involve distinct upstream triggers.

The phospho-ubiquitin model remains speculative. The E3 ubiquitin ligases that recognize LRRK2-phosphorylated Rabs remain unidentified. So do the relevant ubiquitination sites. Direct experimental testing is still awaited. The therapeutic strategies summarized in Table 2 are conceptual or at the earliest preclinical stage. Their clinical translation will require substantial advances in blood–brain barrier penetration, subtype selectivity, and long-term safety profiling.

Finally, studies of Rab trafficking in glial populations and peripheral immune cells remain limited. This is a notable gap. Both cell types shape neurodegenerative outcomes.

## Conclusion

A proteinopathy model centered solely on protein aggregation is not the only way to understand neurodegenerative diseases. We acknowledge the central role of protein aggregation in disease pathophysiology. Even so, this review advances a trafficking-based theory. We propose the term Rabopathy as a disease category for neurological disorders that share one defining feature: progressive dysfunction of Rab GTPase-regulated membrane trafficking. This dysfunction compromises synaptic, endolysosomal, or autophagic flux.

In this model, the nature of the synapse must be reexamined. It is certainly an electrical junction between neurons, but more importantly it is the terminal of a high-fidelity trafficking network. The stability of this network depends on the spatiotemporal coherence of the Rab system. The LRRK2-Rab phosphorylation axis serves as the central homeostatic regulator of this system, and its activity must remain within a narrow physiological window. Once it moves beyond that window, failures cascade through the system. Endosomal congestion and secretory leakage appear first. The process ends in complete stasis of the ALP. RAB39B is a prime example of this developmental-to-degenerative trajectory. A Rab deficit early in life creates a latent vulnerability, and this vulnerability emerges as degeneration only under cumulative stress. From this perspective, the transition from healthy aging to dementia can be understood as the gradual exhaustion of transport reserve. The accumulated Aβ, α-synuclein, and huntingtin are not the initiating causes of disease. They are the catastrophic consequences of a network that has lost its capacity for renewal and clearance. Therapeutic priorities should accordingly shift from clearing extracellular aggregates toward repairing intracellular trafficking, although most strategies targeting this goal remain at the preclinical stage.

Ultimately, the validity of a scientific hypothesis must be decided by experiments. The same holds for the hypothetical phospho-ubiquitin code proposed in this review. Its validation should begin with the identification of the E3 ubiquitin ligases that recognize LRRK2-phosphorylated Rab proteins. This is also the area where a breakthrough is most likely in the near term. Another task is to explain why degeneration strikes only certain neuronal populations. Cell-type-specific Rab interactomes of these vulnerable cells may provide the answer. These insights may eventually lead to intervention. For precision trafficking therapy to enter the clinic, brain-penetrant modulators with selectivity for defined Rab subtypes are indispensable, and their development remains the most technically demanding. The Rabopathy framework remains neurocentric in its current form. Rab GTPases also participate in cancer metabolism and immune cell trafficking. However, the term Rabopathy is restricted here to disorders of the nervous system characterized by synaptic, endolysosomal, or autophagic trafficking failures. Whether similar transport pathologies exist in peripheral tissues is a question that future work will need to address.

## Data Availability

No datasets were generated or analysed during the current study.

## Abbreviations

AD: Alzheimer's disease
AI: Artificial intelligence
ALP: Autophagy-lysosome pathway
AMPA: α-Amino-3-hydroxy-5-methyl-4-isoxazolepropionic acid
APP: Amyloid precursor protein
Arl8b: ADP-ribosylation factor-like 8b
ASO: Antisense oligonucleotide
Aβ: Amyloid-β
BACE1: β-Site APP-cleaving enzyme 1
BDNF: Brain-derived neurotrophic factor
DAT: Dopamine transporter
ER: Endoplasmic reticulum
ESCRT: Endosomal sorting complex required for transport
GABA: γ-Aminobutyric acid
GAP: GTPase-activating protein
GAT: GABA transporter
GDI: GDP dissociation inhibitor
GDP: Guanosine diphosphate
GTP: Guanosine triphosphate
GWAS: Genome-wide association study
HD: Huntington's disease
HIP1: Huntingtin-interacting protein 1
HTT: Huntingtin
IFN-β: Interferon-β
KIF21B: Kinesin family member 21B
LC3B-II: Microtubule-associated protein 1 light chain 3 beta-II
LRRK2: Leucine-rich repeat kinase 2
MSA: Multiple system atrophy
MVB: Multivesicular body
NASPM: 1-Naphthyl acetyl spermine
NMDA: N-methyl-D-aspartate
NMDAR: NMDA receptor
PD: Parkinson's disease
PTM: Post-translational modification
SGK1: Serum- and glucocorticoid-inducible kinase 1
SKIP: SifA and kinesin-interacting protein
SNARE: Soluble N-ethylmaleimide-sensitive factor attachment protein receptor
SV: Synaptic vesicle
TBC1D15: TBC1 domain family member 15
TrkB: Tropomyosin receptor kinase B
VPS: Vacuolar protein sortin
